# The Relationship between Physical Activity Level and Functional Status of Subjects with High Spinal Cord Injury

**DOI:** 10.3390/ijerph19031787

**Published:** 2022-02-04

**Authors:** Ewa Szeliga, Agnieszka Brzozowska-Magoń, Renata Borys, Andżelina Wolan-Nieroda, Katarzyna Walicka-Cupryś

**Affiliations:** Faculty of Health Sciences, Medical Sciences College, University of Rzeszow, Rejtana 16c Street, 35-959 Rzeszow, Poland; brzoza_a@poczta.onet.pl (A.B.-M.); r.borys@vp.pl (R.B.); wolan.a@gmail.com (A.W.-N.); kwcuprys@o2.pl (K.W.-C.)

**Keywords:** spinal cord injury, functional fitness, physical activity, wheelchair rugby

## Abstract

Background. Spinal cord injuries are one of disability in Poland and in the world. Methods: 80 subjects with a transverse injury of the cervical spinal cord were enrolled in the study. The study group included men aged 20–50, 33.1 ± 7.5. A total of 40 (50%) of the subjects comprised the physically active group (AG)—subjects doing wheelchair sport twice a week for 90 min a day. The physically inactive group (IG) comprised 40 (50%) subjects who had not participated in any sports activities. Statistical analyses were carried out using Shapiro-Wilk W-test and Mann-Whitney U test. Results. Significant differences were found between the physically active and inactive men with regard to their functionality status. The biggest differences were found for turning over (*p* < 0.001) and in adopting a sitting position (*p* < 0.001). Persons in the AG group had better results in all assessed activities. The biggest differences were observed in the field of toilet and dressing up: tooth-brushing *p* < 0.007 and washing the top part of the body *p* < 0.002. Conclusions. People participating in regular physical activity–wheelchair rugby–after spinal cord injury have a better relationship with better fitness, greater independence and a better functional status.

## 1. Introduction

Cervical spine injuries with spinal cord injuries are among the most severe con-sequences of accidents, often with a dramatic course. Lack of attention, making a rash decision or an unfortunate coincidence may lead to a tragic accident and, consequently, to disability. Injury to the spinal cord is often included in the category of a “critical life event” that leads to serious changes in almost all areas of human activity and its environment. Problems that result from the inhibition of the spinal cord’s functions, including the inability to move and moving in a wheelchair, as well as the loss of all kinds of sensations, paralysis of the sphincters with involuntary urination and defecation and sexual dysfunction are serious challenges for the individual. They are often a direct source of problems, taking the form of anxiety, depression, psychosomatic and neurotic disorders, and they evoke feelings of shame, helplessness, harm and even alienation in the patient. The scope of changes in physical and physiological functioning and their psychosocial consequences is determined by, i.a. the degree of core damage. Patients react differently to information about their health. At first, they are shocked, an emotion that may be accompanied by despair, anger, rebellion and denial. They do not accept what has happened or what they have to deal with, and they wait for recovery or even a miracle [1].

Due to the high level of damage to the spine along with the disruption of the spinal cord, functional efficiency plays an important role. It conditions the ability to self-service and secures one’s own needs, enabling independent functioning in everyday life.

Being independent of other people is assessed in such activities as preparing and eating meals, controlling physiological functions, maintaining the hygiene of one’s own body, moving, and dressing. Functional efficiency is influenced by health and socio-demographic factors [2]. To perform each of the simplest activities, you need an efficient mind (willingness and ability to perform a given activity), an efficient way of transmitting information (nerve pathways) and the efficiency of the executive system (musculoskeletal system). Even though functional efficiency is mainly a physical process, it also has a psychological dimension, as physical activity deficits adversely affect human well-being and quality of life. Efficient and independent functioning has wide practical and emotional importance for the patient. It has a positive effect on his physical and mental health and fosters independence. People with a severe spinal cord injury are often unable to live independently. Lack of full autonomy makes them need environmental support. The whole family is often involved in caring, and their support is needed around the clock. The daily help a person needs after a spinal cord injury depends on their physical condition, the severity of the spinal cord injury and also their desire to help themselves. One of the most important elements of therapy for a patient with spinal cord injuries, which affects the patient’s functional efficiency, is undertaking physical activity [3].

In the current literature database, there is no research on the comparison of people undertaking regular physical activity against the background of completely inactive people isolated from society. There is also a lack of assessment of fine motor skills in people with a severe spinal cord injury. It is extremely important because it conditions independence in life and allows for independent existence.

Cervical spine injury with spinal cord damage is one of the most serious consequences of accidents, which are often dramatic. The problems resulting from inhibition of the spinal cord function, e.g., inability to move, use of a wheelchair and limited physical performance, are a serious challenge for an individual.

Due to the consequences of spinal cord injury, mainly motor deficits and sedentary lifestyle, much attention is given to physical activity. Movement improves not only the functional status, but also the emotional status [1]. Regular physical activity undertaken by a person with tetraplegia results in improved movement coordination, muscle strength, endurance and fitness, as well as improved fatigue tolerance, which has a significant influence on effective movement in a wheelchair [2]. Improved movement fitness also results in improved everyday functioning, for example, movement between the wheelchair and the bed, tub and car [1,2]. Regular sports activity affects the emotional condition and social life of subjects with spinal cord injuries. Participation in sports and recreation facilitates mental adaptation to life after spinal cord injury, mitigating symptoms of depression and improving an image of oneself [2]. Participation of subjects after spinal cord injury in sports activities may be extremely beneficial with regard to social reintegration, providing an opportunity to make new friends and share experiences. It also develops a network of social support and reduces the feeling of being inferior. Physical activity not only allows keeping a better health state, but also plays an important role in improving physical, mental and social functioning, as well as improving the quality of life and the satisfaction of a disabled person [3,4].

Taking up regular physical activity is a very important element in the life of subjects after spinal cord injury, since it significantly affects their functional status [5]. Participation in physical activity is currently an essential element of complex rehabilitation of subjects after spinal cord injury. A special advantage of physical activity among other rehabilitation forms is the possibility of improving the strength, endurance and fitness of the patient, together with coordination and fatigue tolerance [6]. As well as the motor aspect, physical activity also affects other areas of life, especially emotional state and social life [7]. Engaging in sport facilitates mental adaptation to life after spinal cord injury. It facilitates social integration by providing opportunities to meet people, share experiences and make new friends [6]. Allowing subjects after spinal cord injury to participate in sports and recreation is especially important for reducing the consequences of disability and using their full potential. The achievements of Paralympic athletes are an ideal example of human possibilities [5].

Regular physical activity is recommended for the disabled, especially those who have recently suffered a spinal cord injury [6,7,8]. Participation in various forms of physical activity is not only a condition for better well-being, but also results in improved muscular strength and faster adaptation to disability [9,10,11]. Regular exercise and participation in sports activities contribute to better mental functioning [12,13] as well as to higher levels of life satisfaction and improvement in the quality of life [13,14,15]. Sport is a factor which may affect independence, functional abilities, well-being and professional, social and family life. However, despite these well-known benefits of regular physical activity, participation of subjects after spinal cord injury in such activities is low [16,17,18]. Numerous researchers have shown that the lack of movement of subjects after spinal cord injuries has a great influence on the occurrence of physical and mental health problems. It is also correlated with a lack of self-reliance and reduced quality of life of a disabled person [19,20,21,22,23,24].

The literature review shows no data on the differences among the functional status, health state and quality of life. Moreover, no studies are available on subjects after spinal cord injury who do not take part in any forms of activity, live in social isolation or do not participate in any social and professional life.

The aim of the study was to assess the size of differences in functional fitness between groups of people after severe spinal cord injury.

## 2. Materials and Methods

The study included 80 men after transverse spinal cord injury in the cervical section, men aged 20–50, 33.1 ± 7.5. The study subjects were divided into two groups: physically active and physically inactive. The physically active group included 40 subjects after spinal cord injury who played wheelchair rugby twice a week for 90 min. The physically inactive group included 40 subjects after cervical spinal cord injury who did not take part in any individual or organized forms of physical activity.

Similar sensorimotor deficits were found in all subjects, including no movement or feeling below the damage level. All test persons were able to use an active wheelchair. Most of the surveyed men needed help with the basic functions of everyday life.

The studies were conducted personally with each individual patient in central and southern Poland. All study participants had been informed about the objective of the study and gave their consent for participation before the study started. The study plan was approved by the Ethics Committee.

Study qualification criteria:

- Cervical spinal cord injury at C4-C7;
- Minimum 12 month period since the occurrence of the injury;
- Men aged between 20 and 50;
- Voluntary consent of the patient to conduct the study.

Study exclusion criteria:

- Spinal cord injury below the level of C7;
- Spinal cord injury above the level of C4;
- Time since the occurrence of injury less than 1 year;
- Lack of consent to take part in the study.

The assessment was based on the author’s questionnaire, Konstancin Functional Scale and the WHOQOL-BREF questionnaire.

The WHOQOQ-BREF questionnaire is currently the best tool to evaluate the quality of life, since it allows precise assessment of both subjective and objective indices of quality of life of subjects after spinal cord injury.

The Konstancin Functional Scale (Mazowieckie Rehabilitation Centre “STOCER” Konstancin) defines degrees of independence regarding basic functions of everyday life, i.e., ability to sign, use the computer keyboard, have meals, personal hygiene (brushing one’s teeth, washing the face, washing the top and bottom part of the body), use the toilet, dress up (putting on specific items of clothing: blouse, trousers, socks, gloves), changing position between the bed and using a wheelchair.

Statistical analysis was performed with the use of statistical tests: chi-square test of independence (for determination of the percentage of nominal features), *t*-test for independent samples (to compare average numerical characteristics) and the Mann–Whitney test (for 2 independent groups) (for additional analyses of self-assessment of the health state and quality of life).

## 3. Results

The majority of subjects, 22 (55%), in the physically active group were aged 30–39 years. In the physically inactive group, the highest number of subjects, 9 (22.5%), were aged 25–29 years.

The average age of the study subjects was 33.1 years. In the study population, 25% of men were not more than 27 years old, half of them were no more than 33.5 years old, and 75% were not more than 39 years old. The distribution of the study groups with regard to age is presented in Table 1.

The physically active subjects showed a higher level of self-reliance in daily activities than the physically inactive subjects. For most of the activities (washing and dressing up), the level of self-reliance was significantly higher in the group of active subjects. In the case of brushing one’s teeth (*p* = 0.001) and washing the upper body (*p* < 0.001) the difference was statistically significant, and such a tendency can be noticed in the use of a keyboard (*p* = 0.078), drinking from a cup (*p* = 0.090) and using the toilet (*p* = 0.056). For such activities as signing, holding a sandwich and moving from the bed, no statistically significant differences between the groups were observed, as seen in Table 2.

The mean values for the highest results of the self-reliance study showed significant differences between the active and inactive group (*p* < 0.001), as seen in Table 3.

The assessment included the ability to turn over, change position from the wheelchair onto the bed and to move around without assistance. A statistically significant difference was observed in performing basic activities related to changing position between the bed and wheelchair to the benefit of the physically active group. The biggest differences were observed in turning over (*p* < 0.001) and in adopting a sitting position without assistance (*p* < 0.001), as seen in Table 4.

Subjects from the physically active group left the house without assistance slightly more often (67.5%) than the physically inactive subjects (55%). Differences between the groups were not statistically significant, as seen in Table 5.

## 4. Discussion

For subjects after spinal cord injury, it is very important to achieve the best possible functional level, which affects their whole life. To prepare them for active life in society, with family and at work, a very important role is played by camps and activities organized for active rehabilitation [25]. Participation in such activities allows achieving a high level of movement and self-reliance abilities of subjects with a spinal cord injury. Instructors are usually people with the same disability. They guide the patient and inspire them by having the same disability, leading active life and being a rich source of human experience, both with regard to specific skills and information about the rights, obligations and possibilities of subjects after spinal cord injury [14,26].

People who participated in wheelchair rugby obtained significantly better results in terms of fine and gross motor activities such as doing up buttons, putting on gloves, putting on socks, putting on trousers, putting on a blouse, washing the bottom part of the body, washing the top part of the body and other activities related to toileting and everyday functioning than physically inactive people. Similar results were achieved by Ravenek, Ginis, Smerjian and Tasiemski and Van der Ploeg, who studied subject taking part in regular physical activity after spinal cord injury, for which they found a higher level of subject independence and observed that physically active subjects are more self-reliant in their daily activities [27,28,29,30]. The studies by Ginis et al., similarly to our studies, showed improved functional independence in various daily activities and movement in the group of physically active subjects after spinal cord injury [31,32,33]. Additionally, studies by Fliess-Douer confirm that physical activity significantly improves functional status and, as a result, movement [34]. Similar results were presented by Lipert, emphasizing a positive influence of movement and active rehabilitation camps on functional status (with regard to daily activities and movement) of subjects after cervical spinal cord injury [35]. Our own results are different from the results achieved by Nas, where only a slight improvement was observed in such activities as dressing up and using the toilet without assistance [26].

Regular physical activity is recommended for the disabled, especially those with a spinal cord injury. [6,7,8]. Participation in various forms of physical activity is not only a condition for better well-being, but also results in improved muscular strength and faster adaptation to disability [9,10,11]. Regular exercise and participation in sports activities contribute to better mental functioning, [12,13] as well as to higher levels of life satisfaction and improvement in the quality of life [13,14,15]. However, despite these well-known benefits of regular physical activity, participation of subjects after spinal cord injury in such activities is low [17,18]. Sport is a factor which determines their independence, functional abilities and well-being, as well as their professional, social and family life.

People after spinal injury often encounter numerous barriers preventing them, or making it impossible, to become involved in physical activity and an active social life. They are related to costs of travelling to the training site, sports activities and architectural barriers [36,37,38,39,40,41,42]. Due to these problems, persons after spinal cord injuries often decline to take up physical activity. Studies of Tasiemski and Anneken show that as high as 50% of the population of subjects after spinal cord injury is physically inactive [20,30]. The overlapping factors may lead to further disability, contributing to reduced mobility and fitness, as well as to reduced ability to perform activities of daily living, and finally to complete dependence on others and a reduced quality of life of subjects after spinal cord injury [28,43].

Taking up regular physical activity—in contrast to physically inactive subjects—is an important factor which has a positive effect on the life of subjects after spinal cord injury. It is very important to establish a broad understanding of the quality of life and attitude towards a disability. Patients working in a team with other people with the same disability not only sooner but also more easily adapt to the new, difficult situation. Sport is not only a form of recreation, but also rehabilitation. Regular physical activity clearly differentiates between the groups of physically active and inactive subjects, which has been concluded here.

## 5. Conclusions

1. There was a difference in physical activity between rugby practitioners and non-rugby practitioners, in favor of rugby practitioners.
2. Due to their low functional fitness, physically inactive subjects remain dependent on third persons, and are unable to exist on their own.
3. Rugby training is recommended for people with spinal cord injury because of the positive physical and mental effects.

## Figures and Tables

**Table 1:** The distribution of the study groups with regard to age.

Age [in Years]	Active Subjects	(%)	Inactive Subjects	(%)	Total N	(%)
20–24	5	12.5	6	15.0	11	13.6
25–29	7	17.5	9	22.5	16	20.0
30–34	11	27.5	6	15.0	17	21.3
35–39	11	27.5	6	15.0	17	21.3
40–44	5	12.5	8	20.0	13	16.3
45–49	1	2.5	5	12.5	6	7.5
Total	40	100	40	100	80	100
*p*	0.249					

**Table 2:** Self-reliance in performing daily activities.

Activities	Group				*p*
	Active Subjects		Inactive Subjects		
	*N*	%	*N*	%	
manual signing	40	100	39	97.5	0.314
using a keyboard	40	100	37	92.5	0.078
teeth brushing	40	100	30	75.0	0.001
holding a sandwich	40	100	38	95.0	0.152
drinking from a cup	39	97.5	35	87.5	0.090
moving between the bed and wheelchair	31	77.5	26	65.0	0.217
using the toilet	31	77.5	23	57.5	0.056
washing the top part of the body	38	95.0	24	60.0	<0.001

**Table 3:** Distribution of the study groups with regard to assessment of self-reliance.

Group	Assessment of Self-Reliance (Score)				
	$\bar{\chi }$	Me	*S*	Min	Max
active subjects	8.6	10.0	2.3	3.0	10.0
inactive subjects	5.3	5.5	2.9	0.0	10.5
*p*	<0.001				

**Table 4:** Distribution of the study groups with regard to basic activities related to changing position.

Performing Basic Activities	Group				*p*
	Active Subjects		Inactive Subjects		
	*N*	%	*N*	%	
turning over	29	72.5	5	12.5	<0.001
sitting on the bed	30	75.0	10	25.0	<0.001
sitting with legs hanging down the bed	29	72.5	16	40.0	0.003

**Table 5:** Distribution of the study groups with regard to leaving the house without assistance.

Going Out without Assistance	Group				Total	%
	Active Subjects	%	Inactive Subjects	%		
Yes	27	67.5	22	55.0	49	61.5
No	13	32.5	18	45.0	31	38.5
Total	40	100	40	100	80	100
*p*	0.251					

## Data Availability

The datasets used and/or analyzed in the present study are available from the respective author—Ewa Szeliga—upon reasonable request.
